# Bile acid-independent protection against *Clostridioides difficile* infection

**DOI:** 10.1371/journal.ppat.1010015

**Published:** 2021-10-19

**Authors:** Andrea Martinez Aguirre, Nazli Yalcinkaya, Qinglong Wu, Alton Swennes, Mary Elizabeth Tessier, Paul Roberts, Fabio Miyajima, Tor Savidge, Joseph A. Sorg

**Affiliations:** 1 Department of Biology, Texas A&M University, College Station, Texas, United States of America; 2 Baylor College of Medicine & Texas Children’s Hospital, Houston, Texas, United States of America; 3 Royal Liverpool and Broadgreen University Hospitals NHS Trust, Liverpool, United Kingdom; 4 Oswaldo Cruz Foundation, Ceara branch, Fortaleza, Brazil; University of Pittsburgh School of Medicine, UNITED STATES

## Abstract

*Clostridioides difficile* infections occur upon ecological / metabolic disruptions to the normal colonic microbiota, commonly due to broad-spectrum antibiotic use. Metabolism of bile acids through a 7α-dehydroxylation pathway found in select members of the healthy microbiota is regarded to be the protective mechanism by which *C*. *difficile* is excluded. These 7α-dehydroxylated secondary bile acids are highly toxic to *C*. *difficile* vegetative growth, and antibiotic treatment abolishes the bacteria that perform this metabolism. However, the data that supports the hypothesis that secondary bile acids protect against *C*. *difficile* infection is supported only by *in vitro* data and correlative studies. Here we show that bacteria that 7α-dehydroxylate primary bile acids protect against *C*. *difficile* infection in a bile acid-independent manner. We monoassociated germ-free, wildtype or *Cyp8b1*^*-/-*^ (cholic acid-deficient) mutant mice and infected them with *C*. *difficile* spores. We show that 7α-dehydroxylation (*i*.*e*., secondary bile acid generation) is dispensable for protection against *C*. *difficile* infection and provide evidence that Stickland metabolism by these organisms consumes nutrients essential for *C*. *difficile* growth. Our findings indicate secondary bile acid production by the microbiome is a useful biomarker for a *C*. *difficile*-resistant environment but the microbiome protects against *C*. *difficile* infection in bile acid-independent mechanisms.

## Author summary

Secondary bile acid production by the colonic microbiome strongly correlates with an environment that is resistant to *C*. *difficile* invasion. However, it remained unclear if these bile acids provided *in vivo* protection. Here, we show that members of the microbiome that generate secondary bile acids (*e*.*g*., *C*. *scindens*) protect against *C*. *difficile* disease independently of secondary bile acid generation. These results are important because efforts to restore colonization resistance (*e*.*g*., FMT or precision bacterial therapy) focus on restoring secondary bile acid generation. Instead, restoring the organisms that produce 5-aminovalerate or consume proline / glycine are more important.

## Introduction

The Centers for Disease Control and Prevention estimates that over 223,000 *Clostridioides difficile* (formerly *Clostridium*) infections (CDI) require hospital care and 13,000 of those cases resulted in fatality [1]. Moreover, the CDC has classified *C*. *difficile* as an urgent threat to the US healthcare system because of the emergence of antibiotic resistant strains [2–4]. Due to the strictly anaerobic nature of *C*. *difficile* vegetative cells, the spore form is required for host-to-host transmission [5,6]. Once ingested, these metabolically-dormant spores germinate in response to certain host-derived bile acids and certain amino acids [7–10].

**Fig 1:**
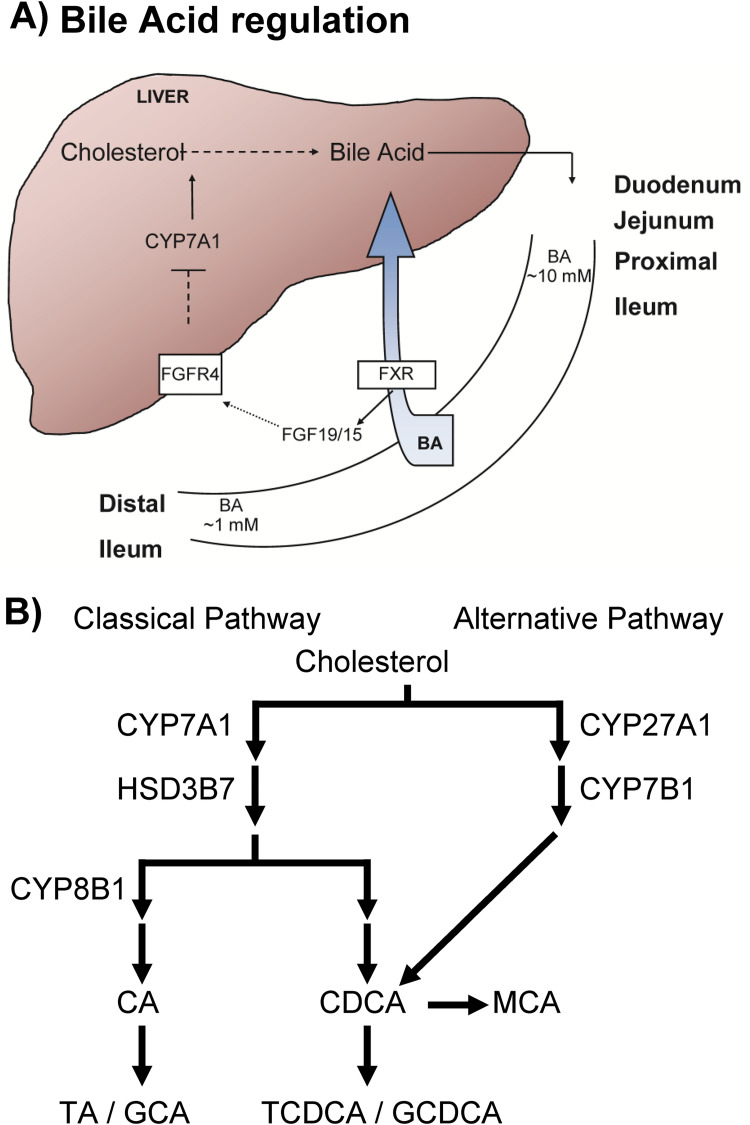
Bile acid regulation. **A**) Bile acids are synthesized in the liver and secreted into the GI at a concentration of nearly 10 mM. During GI transit, approximately 90% is actively reabsorbed and recycled into the liver to be used in new rounds of digestion. During this recycling, bile acids signal through FXR, FGF19/15 and FGFR4 to regulate the rate limiting step of bile acid synthesis, CYP7A1. **B**) Bile acids are synthesized in two pathways. In the alternative pathway, only CDCA (chenodeoxycholate) derivatives (in humans) and CDCA / MCA (muricholate) derivatives (in mice) are made. In the classical pathway, both CDCA and CA (cholate) derivatives are formed. CA synthesis is dependent on CYP8B1 and the absence of CYP8B1 leads to only CDCA (and MCA) formation.

Broad-spectrum antibiotic use is the greatest risk factor for CDI due to the disruption of the colonic microbiome that mediates colonization resistance against this pathogen [11]. Antibiotics lead to dramatic alterations to the colonic metabolome and, importantly, bile acid profiles [12–14]. Bile acids are steroid-like molecules that are synthesized in the liver using cholesterol as a precursor and, when secreted into the gut, aid in the absorption of fats and cholesterol [15]. In the liver, the rate limiting step of bile acid synthesis is the cytochrome P450 7A1 (CYP7A1) enzyme which catalyzes the addition of 7α-hydroxyl to cholesterol [15] and is critically regulated by farnesoid X receptor (FXR) and downstream FGF19 signaling (Fig 1A). Later in the bile acid synthesis pathway, cholic acid (CA) is generated by CYP8B1 adding a 12α-hydroxyl group [15]. In the absence of CYP8B1 activity, only chenodeoxycholic acid (CDCA) derivatives are generated [15,16]. These two major bile acids are then further modified by conjugation of an amino acid (*i*.*e*., taurine or glycine) to the carboxyl (Fig 1B) [15].

Cholic acid-class bile acids activate *C*. *difficile* spore germination [8,10] and chenodeoxycholic acid-class bile acids are competitive inhibitors of cholic acid-mediated spore germination [8,9,17,18]. *C*. *difficile* spores can use different amino acids as cogerminants with cholic acid-derivatives to stimulate spore germination, with varying efficiencies [7]. Bile acid germinants are recognized by the CspC pseudoprotease germinant receptor [8] and the cogerminants (*e*.*g*., glycine) are recognized by the CspA pseudoprotease germinant receptor [19]. Recent work has shown that glycine is an important *in vivo* spore cogerminant and consumption of glycine by one *C*. *difficile* strain prevents spore germination (and colonization) by an invading strain [20].

During digestion, most of the bile acid pool is reabsorbed by the gut and recycled to the liver to aid in new rounds of digestion [21]. However, approximately 10% of the total bile acid pool escapes enterohepatic recirculation and enters the colon where microbiota deconjugate the taurine or glycine from the primary bile acid [21]. Subsequently, the deconjugated bile acids are taken up by a few members of the normal, healthy, microbiota where they are metabolized to generate secondary bile acids. In a series of enzymatic steps, these bacteria remove the 7α-hydroxyl group to generate deoxycholate (DCA) from CA and lithocholate (LCA) from CDCA [21]. The generation of these secondary bile acids is hypothesized to protect against the invasion by several human pathogens [22–25].

It is well-established that a microbiota containing secondary bile acid-producing bacteria (*e*.*g*., *Clostridium scindens*, a member of Clostridium Cluster XIVa) and that is dominated by secondary bile acids is an environment that resists *C*. *difficile* invasion [12,26–30]. The prevailing model is that the generation of secondary bile acids prevents *C*. *difficile* colonization due to the toxicity of these bile acids towards *in vitro C*. *difficile* vegetative growth (S1A Fig) [12,26,29,31–35]. Antibiotic treatment of healthy hosts eliminates both secondary bile acid production and the bacteria that produce these molecules (S1B Fig) [12,26]. Interestingly, fidaxomicin is a narrow spectrum antibiotic approved to treat CDI and has a lower recurrence rate than other antibiotics [36]. Importantly, fidaxomicin does not target members of Clostridium Cluster XIVa, further highlighting the importance of these organisms in the prevention of CDI [37]. Despite these studies, the data that support the hypothesis that secondary bile acid production and their *in vivo* toxicity towards *C*. *difficile* growth only are supported by correlative studies.

Here, we test the impact of cholic acid-class bile acids on the initiation of *C*. *difficile* disease and how members of the colonic microbiota that can metabolize primary bile acids to secondary bile acids protect against CDI in the absence of cholic acid. Our results suggest that secondary bile acid generation is dispensable for providing protection against *C*. *difficile* invasion and that the microbiome protects against CDI by consuming nutrients that are important for *C*. *difficile* colonization.

## Results

### *Bai-*encoding bacteria protect against *C*. *difficile* infection

**Fig 2:**
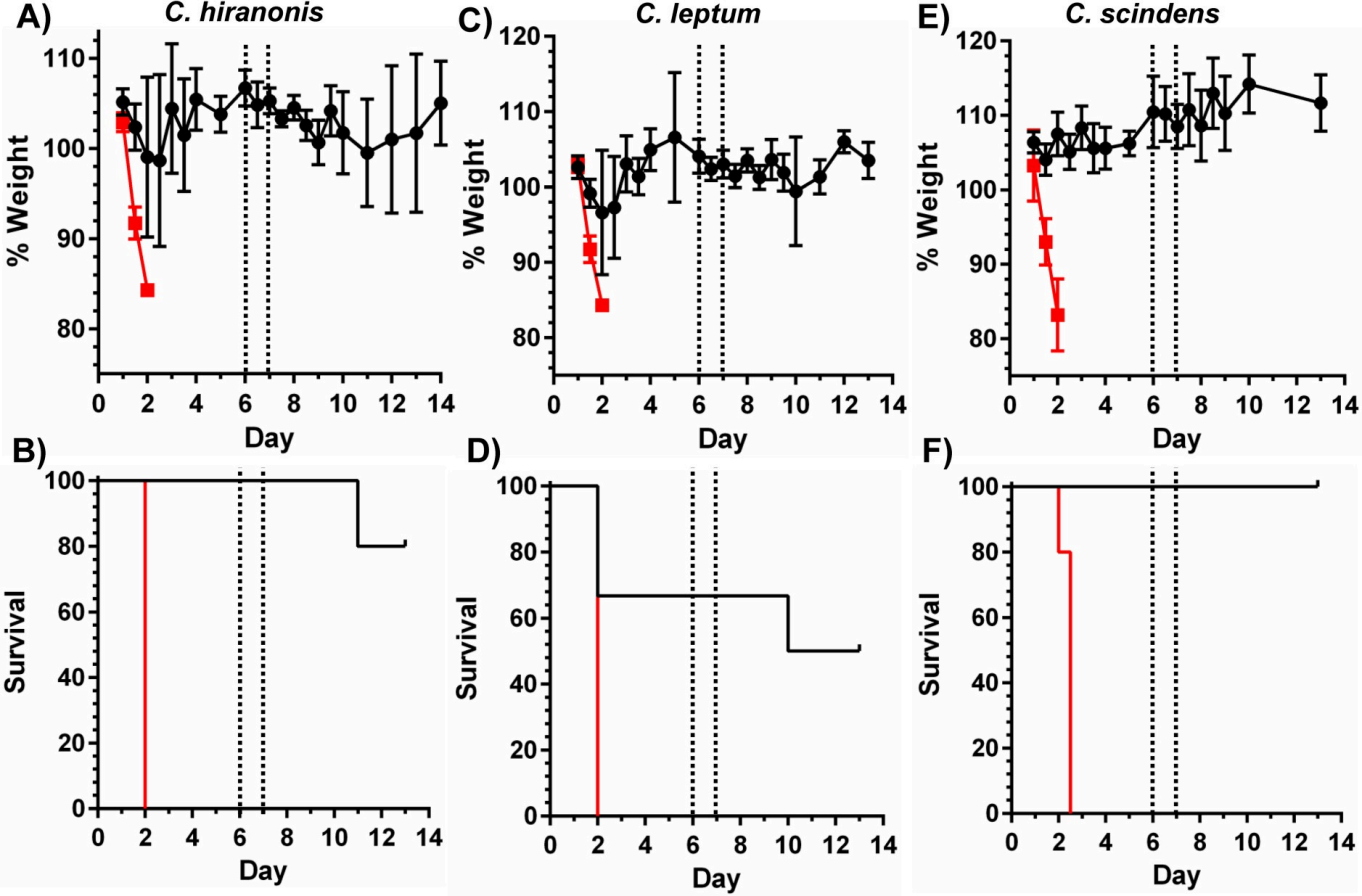
Monoassociation of germ-free mice with bile acid metabolizing bacteria protects against CDI. Monoassociation of germ-free mice with (A) and (B) *C*. *hiranonis* 10542 *(*N = 6), (C) and (D) *C*. *leptum* ATCC29065 *(*N = 6) or (E) and (F) *C*. *scindens* VPI12708 *(*N = 11) protects mice against CDI. Black line denominates monoassociated mice, red line denominates germ-free mice. On days 6 and 7 post CDI, monoassociated mice were given a single i.p. dose of clindamycin to induce recurrence [39]. A, C and E represent the average daily weights of the infected mice. B, D and F represent the Kaplan-Meier survival.

To investigate if secondary bile acid production by the microbiota is essential for establishing an environment that resists *C*. *difficile* invasion, we monoassociated germ- free mice with either *Clostridium hiranonis* 10542, *Clostridium leptum* ATCC29065, or *C*. *scindens* VPI12708. These organisms have been reported to metabolize deconjugated primary bile acids and generate secondary bile acids [21,38]. We first confirmed that these organisms encode an orthologue of the bile acid 7α-dehydroxylation pathway, the 7α-dehydratase, *baiE* (S2 Fig) [21]. Germ-free or mice monoassociated with the indicated strain were infected with 10^4^ *C*. *difficile* VPI10463 spores, a highly pathogenic *C*. *difficile* strain that is also as sensitive to bile acids as other *C*. *difficile* strains (S1 Table) [9]. *C*. *hiranonis* or *C*. *leptum* monoassociated mice were protected against infection, and did not show significant clinical symptoms until clindamycin was administered to induce disease recurrence (Fig 2A–2D) [39]. Notably, *C*. *scindens*-monoassociated mice were completely protected against primary or recurrent CDI, with mice showing no signs of disease (Fig 2E and 2F). Quantitative analysis of bile acid profiles in these monoassociated mice, demonstrated very little secondary bile acids compared to large amounts of primary bile acids (S3 Fig and S2 Table). This is likely due to the production of conjugated bile acids by the host and the inability of these organisms to deconjugate the bile acids prior to 7α-dehydroxylation [21,40]. However, in one *C*. *hiranonis*-colonized mouse, we observed CA present and in one *C*. *scindens*-colonized mouse DCA was present, suggesting that *C*. *hiranonis* and *C*. *scindens* may have deconjugated TA to CA. Thus, we tested the ability of *C*. *hiranonis* and *C*. *scindens* to deconjugate TA (S4 Fig). When *C*. *scindens* and *C*. *hiranonis* were grown for 24 hours in medium supplemented with TA, we did not observe *C*. *scindens*-mediated deconjugation of TA to CA indicating that C. scindens VPI12708 does not deconjugate primary bile acids. However, *C*. *hiranonis* did deconjugate the majority of TA to CA. This in vitro data thus suggest that in the *C*. *hiranonis*-colonized mouse the presence of CA could be due to deconjugation of TA by the bacterium. Conversely, because *C*. *scindens* could not deconjugate TA to CA, the DCA found in the *C*. *scindens*-colonized mouse likely was due to a small amount of CA made by the host that was then 7α-dehydroxylated by *C*. *scindens*.

### Absence of cholic acid does not impact the colonic microbiome

Despite the small amount of secondary bile acids produced in the monoassociated mice, we wanted to eliminate the production of the predominant secondary bile acid, DCA, proposed to exert protection against CDI. *Cyp8b1*^-/-^ mice do not synthesize CA and, thus, the precursor for DCA production by the microbiota is not present. Li-Hawkins *et al*. [16] first described this animal model and heterozygous / homozygous mice are indistinguishable from the wildtype strain. Bile acid analysis of conventionally raised, wildtype, heterozygous and homozygous mutant mice revealed that fecal samples from both wildtype and heterozygous mice were dominated by the primary bile acids taurocholate (TA) and β-muricholate (BMCA; a CDCA derivative found in mice that is also toxic to *C*. *difficile* vegetative growth but the concentration found in these mice is below the minimal inhibitory concentration [9]) and the secondary bile acids DCA and LCA (Table 1). However, fecal samples from *Cyp8b1*^-/-^ mice did not contain CA-derivatives or secondary bile acids (Table 1) as reported previously [16].

**Table 1:** Bile acid profiles of conventionally raised wildtype, CYP8B1 heterozygous and CYP8B1 homozygous mice.

	Concentration (nmol / g)						
	Primary Bile Acids				Secondary Bile Acids		
	TA	CDCA	AMA	BMA	DCA	LCA	OMA
Wildtype	26.9	-	12.7	29.8	-	-	-
Wildtype	34.4	-	-	6.4	33.5	62.0	10.4
CYP8B1 HET	108	13.7	-	18.8	-	55.6	-
CYP8B1 HET	34.9	-	-	12.1	83.9	-	111.3
CYP8B1 HOM	-	-	151.5	50.4	-	-	-
CYP8B1 HOM	-	18.0	-	41.0	-	-	-

taurocholate (TA), chenodeoxycholate (CDCA), alpha-muricholate (AMA), beta-muricholate (BMA), deoxycholate (DCA), lithocholate (LCA), omega-muricholate (OMA)

below the limit of detection

Limit of detection for Sedere Sedex model 80 LT- ELSD was calculated to be 0.2 nmol.

GCA, CA, TCDCA, GCDCA were below the limit of detection

**Fig 3:**
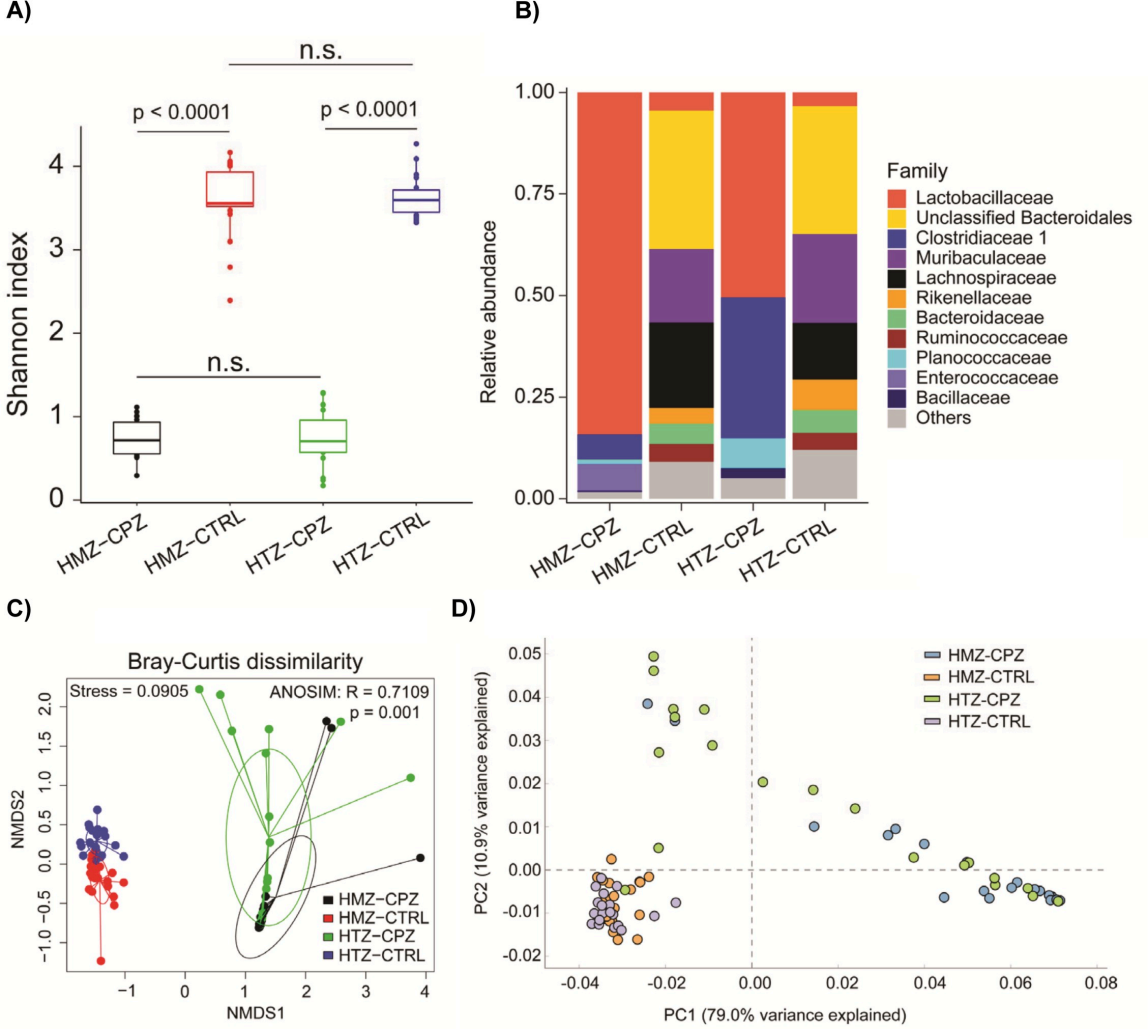
Microbiome analyses of *Cyp8b1*^*+/-*^ and *Cyp8b1*^*-/-*^ mice. A) Alpha-diversity analysis with Shannon index of fecal microbiome of heterozygous(N = 5) (HTZ) and homozygous (HMZ) (N = 5) mice treated with or without cefoperazone (CPZ)(N = 5); two tailed Mann-Whitney test was used for two-group comparison; B) Taxonomic abundance at family rank for mouse fecal microbiome; C) Beta-diversity analysis with Bray-Curtis dissimilarity distance metric and non-metric multidimensional scaling (NMDS); non-parametric statistical test for group comparisons was conducted with analysis of similarities (ANOSIM) method; D) Principal component analysis for PICRUSt2-based metabolic pathway abundance inferred from 16S amplicon data.

The loss of a major bile acid class could greatly impact the colonic microbiome, pre- or post-antibiotic treatment. To understand this, we collected fecal samples from *Cyp8b1*^+/-^ and *Cyp8b1*^*-/-*^ mice and their microbiome profiles were determined. Interestingly, these mice had no significant differences in their microbiome and, when treated with the broad-spectrum antibiotic, cefoperazone, their microbiomes were equally disrupted (Fig 3A–3D). These results indicate that the loss of one major bile acid class does not adversely affect host microbiome composition.

### Bile acid-mediated germination is not essential for infection in germ-free mice

**Fig 4:**
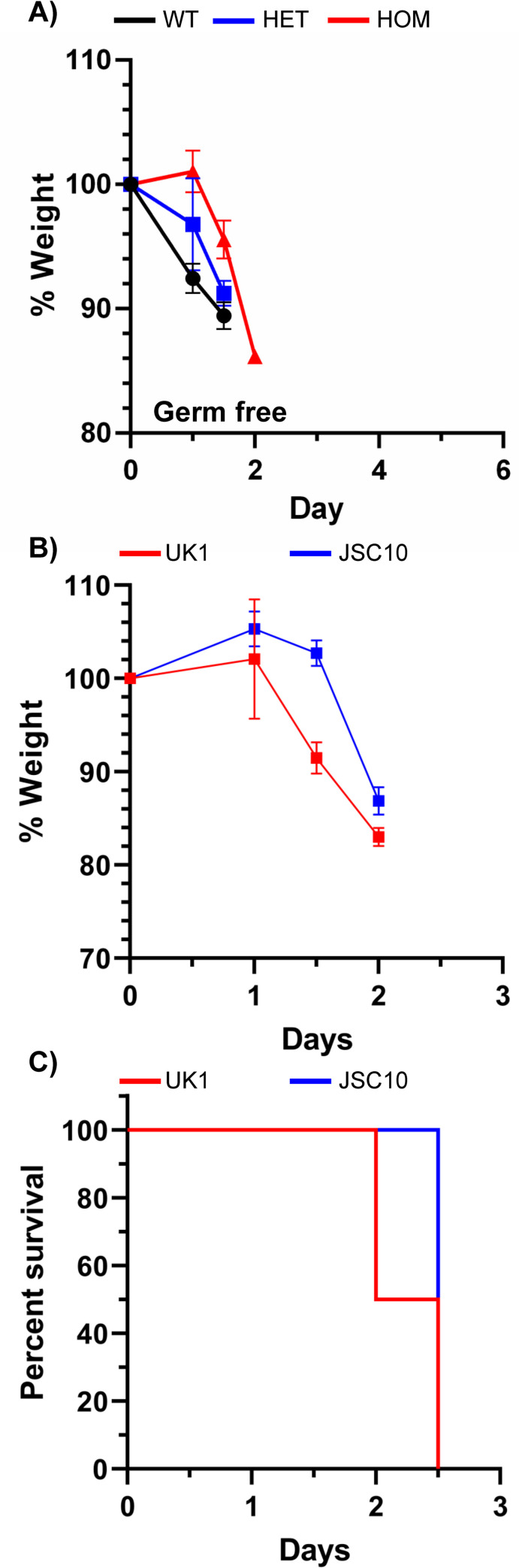
Cholic acid derivatives are not required to initiate *C*. *difficile* infection. **A**) Germ-free wild type (N = 3), CYP8B1 +/- (N = 3) or CYP8B1 -/- (N = 3)were infected with 10^5^ *C*. *difficile* VPI10463 spores and monitored for signs of disease. Weight loss (**B**) or Kaplan-Meier survival plot (**C**) of CYP8B1 -/- mice infected with wildtype *C*. *difficile* UK1 or *C*. *difficile* JSC10 (*cspC*::*ermB*).

Bile acids are essential for *in vitro* germination of *C*. *difficile* spores [8,10] and signal through the CspC germinant receptor [41], with taurocholic acid being the primary activating ligand. In a hamster model of CDI, spores derived from a *C*. *difficile cspC*::*ermB* (JSC10) mutant strain were still partially virulent, indicating that some basal level of spontaneous spore germination occurs *in vivo* [8]. Because the *Cyp8b1*^*-/-*^ strain does not synthesize CA derivatives (*i*.*e*., taurocholate) we tested the importance of bile acid- mediated germination from the host side. Germ-free wildtype, *Cyp8b1*^*+/-*^, and *Cyp8b1*^*-/-*^ mice were infected with *C*. *difficile* VPI10463 spores. Surprisingly, despite the absence of a potent *C*. *difficile* spore germinant (taurocholic acid), *C*. *difficile* spores germinated and caused disease equally in the homozygous and heterozygous strains (Fig 4A).

We next tested if the disease we observed for the *Cyp8b1*^-/-^ strain was due to an autogermination process of the inoculated spores, as we reported in prior work [8]. We infected *Cyp8b1* homozygous mutant mice with spores derived from *C*. *difficile* JSC10 (*cspC*::*ermB*) and the *C*. *difficile* UK1 parental strain. Because the bile acid germinant receptor is inactivated in this strain, spores are unable to respond to bile acids and, instead, germinate in a bile acid-independent manner. Infection of germ-free *Cyp8b1*^*-/-*^ mice with spores derived from the *C*. *difficile* JSC10 strain resulted in identical clinical symptoms (Fig 4B), and fulminant disease as the *C*. *difficile* UK1 strain (Fig 4C). These results suggest that at the dose used for these mouse experiments (10^6^ spores), bile acid-mediated germination plays little role in colonization and disease pathogenesis.

### Secondary bile acids are not essential to protect against *C*. *difficile* infection

**Fig 5:**
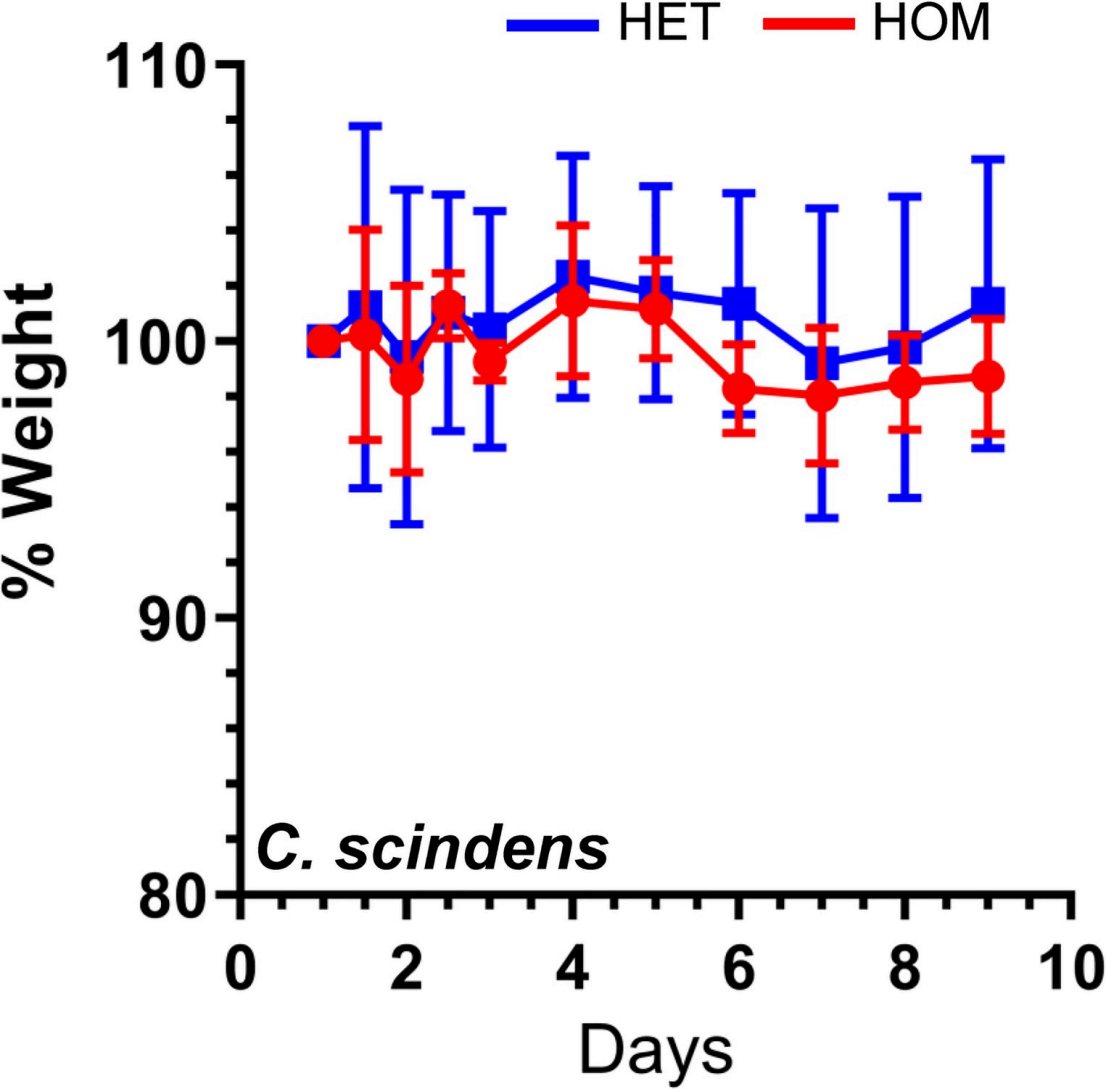
Cholic acid derivatives are not required for protection against CDI. *C*. *scindens*-monoassociated CYP8B1 +/- (N = 3) and CYP8B1 -/- (N = 4) mice were infected with *C*. *difficile* VPI10463 and monitored for signs of disease.

Next, we monoassociated germ-free heterozygous and homozygous *Cyp8b1* mutant mice with *C*. *scindens*. Analysis of colonization levels indicated that *C*. *scindens* monoassociation was not perturbed in the absence of CA synthesis by the host (S3 Table). Oral administration of *C*. *difficile* VPI10463 spores resulted in no disease in *C*. *scindens* colonized animals (Fig 5), whereas germ-free *Cyp8b1* mice rapidly succumbed to infection irrespectively of genotype (Fig 4A). Analysis of colonization levels 4 days post infection demonstrated that *C*. *scindens* abundance was reduced with *C*. *difficile* still being detectable (S3 Table). This suggests either that *C*. *scindens* or *C*. *difficile* compete for niche or nutrients during colonization, or that secondary bile acids have accumulated and hinder *C*. *difficile* growth. To understand their bile acid profiles, we extracted cecal samples from *C*. *scindens* monoassociated heterozygous or homozygous mice (Table 2). As expected, no cholate derived secondary bile acids were observed in *C*. *scindens* colonized *Cyp8b1*^-/-^ mice before, whereas heterozygous animals possessed some DCA in the presence of *C*. *difficile* (Table 2). Because we did not observe differences in protection between *C*. *scindens* monoassociated *Cyp8b1*^-/-^ and *Cyp8b1*^+/-^ strains, production of secondary bile acids have no or minimal impact in establishing colonization resistance and host protection occurs in a bile acid-independent manner.

**Table 2:** Bile acid profiles of *C*. *scindens* colonized CYP8B1 heterozygous and CYP8B1 homozygous mice.

	Concentration (nmol / g)					
	Primary Bile Acids			Secondary Bile Acids		
	TA	CA	BMA	DCA	LCA	OMA
CYP8B1 HET	1.0	-	10.0	39.7	-	92.1
CYP8B1 HET	7.1	21.2	44.1	24.9	-	-
CYP8B1 HET	-	18.2	115.1	27.4	-	-
CYP8B1 HOM	-	-	4.0	-	-	6.7
CYP8B1 HOM	-	-	39.3	-	-	-
CYP8B1 HOM	-	-	6.9	-	-	-

taurocholate (TA), chenodeoxycholate (CDCA), alpha-muricholate (AMA), beta-muricholate (BMA), deoxycholate (DCA), lithocholate (LCA), omega-muricholate (OMA)

below the limit of detection

Limit of detection for Sedere Sedex model 80 LT- ELSD was calculated to be 0.2 nmol.

GCA, TCDCA, GCDCA, CDCA, AMA were below the limit of detection

### Cholic acid-class bile acids are not essential for successful FMT

**Fig 6:**
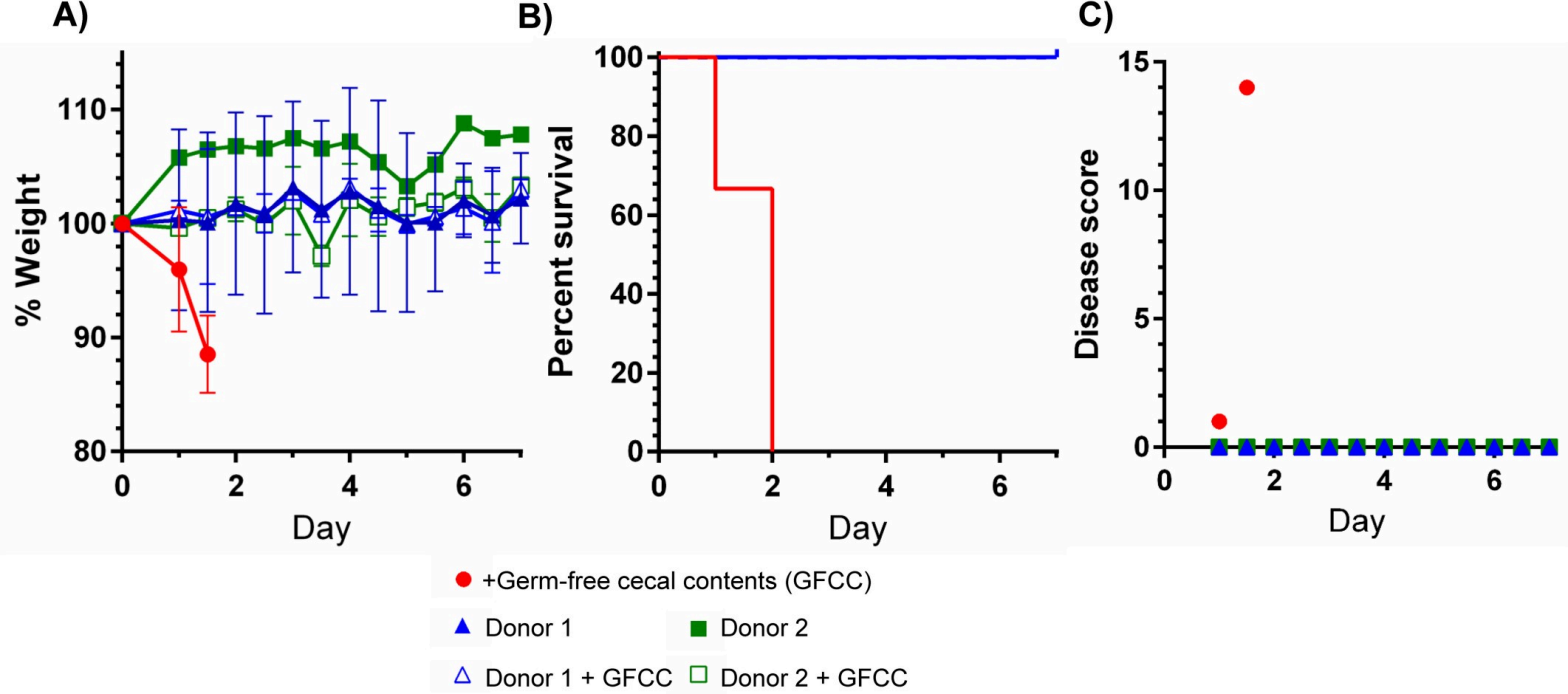
Fecal microbial therapy protects mice independent of cholic acid class bile acids. Germ-free *Cyp8b1*^*-/-*^ given cecal contents derived from a germ-free donor (GFCC) (N = 3) or mice colonized with human stool donor #1 with (N = 3) or without GFCC (N = 3) and human stool donor #2 with (N = 2) or without GFCC (N = 1) were infected with 10^4^ spores derived from the *C*. *difficile* VPI10463 strain. Weight loss (A) or Kaplan-Meier survival curve (B) or disease scores (C) are illustrated.

Direct infusion of a healthy microbiota into a dysbiotic colonic environment is well established to restore colonization resistance. This fecal microbial therapy (FMT) is thought to provide a quick restoration of the diversity needed to provide colonization resistance to the host. Again, the most commonly stated mechanism is that the FMT restores the production of secondary bile acids (*e*.*g*., DCA) that hinders the establishment of *C*. *difficile* in the gut [42]. To understand if FMT can protect against CDI in *Cyp8b1*^*-/-*^ mice, we inoculated these germ-free mice with human donor stool preparations used in clinical FMT procedures and assessed if the resulting humanized microbiota was still protective in the absence of bile acids (Fig 6). Germ-free *Cyp8b1*^*-/-*^ mice infused with germ-free cecal content were not protected against CDI. However, infusion of stool from two different healthy FMT donors (either directly or as a slurry with germ-free cecal content as a control) completely protected the *Cyp8b1*^*-/-*^ from disease (Fig 6A–6C). These results demonstrate translational relevance by showing that cholic acid-class bile acids are dispensable for successful FMT using human microbiota.

### Stickland metabolites are altered in monoassociated mice

Based on our findings that secondary bile acid production is not the mechanism by which bile acid metabolizing bacteria protect against CDI, we explored whether silencing of downstream FXR signaling could account for host protection. Using conventional FXR^-/-^ mice, we demonstrated that a similar CDI disease course was evident in FXR-depleted and wild type littermates orally administered *C*. *difficile* VPI10463 (S5 Fig). Furthermore, FXR signaling did not appear to be significantly altered in patients with active CDI disease since no differences were evident in downstream serum FGF-19 levels when compared with age-matched hospitalized controls without diarrhea (S6 Fig). Surprisingly, this finding was made despite measuring significantly altered primary and secondary bile acid profiles that are associated with CDI susceptibility in patients (S6 Fig).

To discover alternate bile-acid independent disease mechanisms, we performed unbiased global metabolomics on fecal samples from CDI patients and in mice monoassociated with *C*. *scindens*, *C*. *hiranonis*, *C*. *leptum*, and conventional and germ- free controls. A prior report found that *C*. *difficile* and *C*. *scindens* compete *in vitro* [43]. In this report *C*. *scindens* was found to produce and secrete the tryptophan-derived antibiotic 1-acetyl-β-carboline [43]. However, 1-acetyl-β-carboline alone did not inhibit *C*. *difficile* growth unless added at high concentrations and required synergy with secondary bile acids to confer significant anti- *C*. *difficile* activity. Our metabolomics interrogation using purified standards did not measure detectable 1-acetyl-β-carboline in fecal specimens from *C*. *scindens* monoassociated mice or in healthy subjects who are resistant to *C*. *difficile* colonization. We confirmed these findings by targeted selected-reaction monitored mass spectrometry (sensitivity >1 nM in fecal specimens), indicating that production of this tryptophan-based antibiotic is likely not contributing to protection in our animal models or in patients.

**Fig 7:**
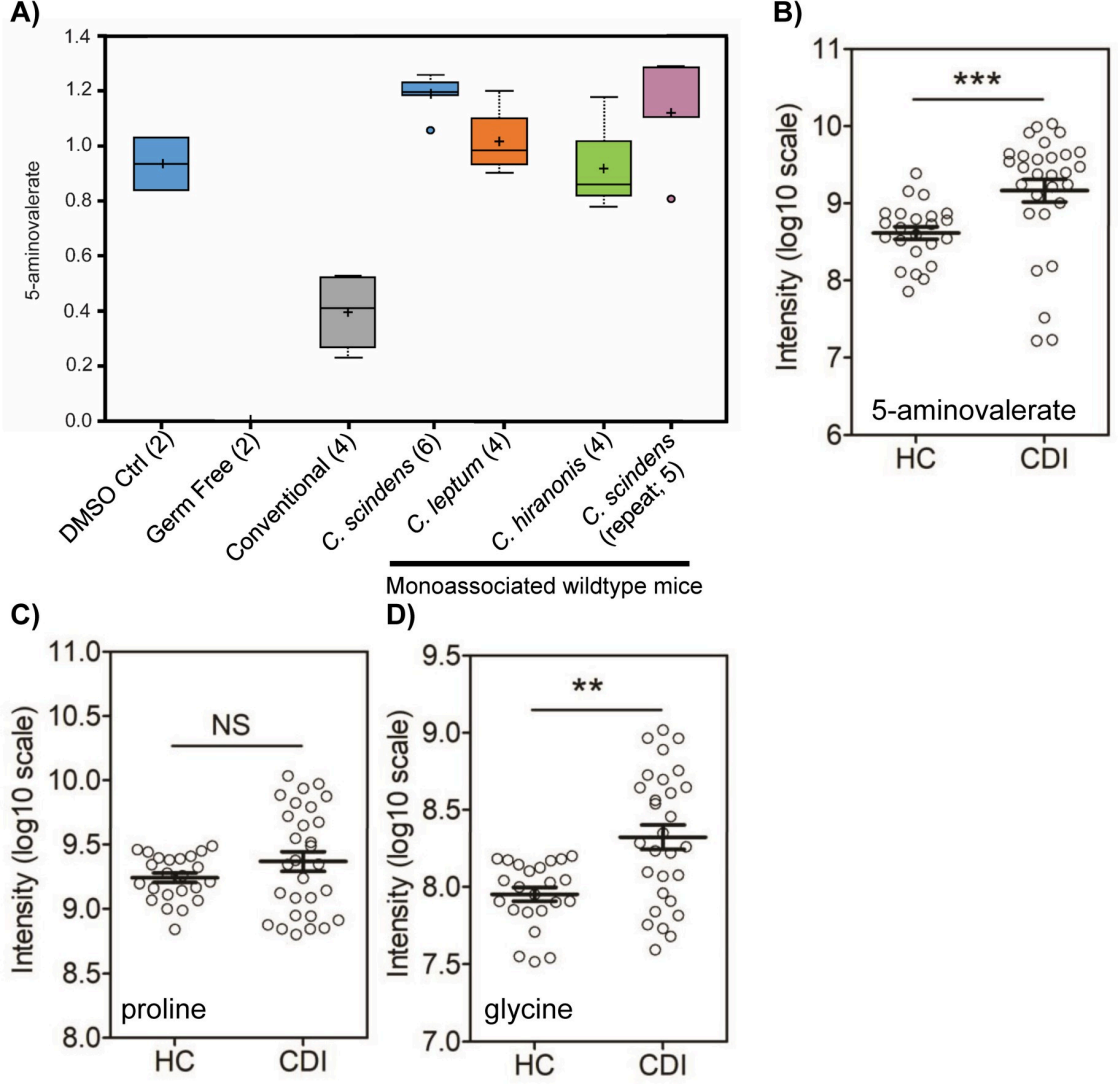
Untargeted metabolomics implicates Stickland metabolism / metabolites in colonization resistance. A) Cecal contents from the indicated mouse samples (number of samples in parentheses) were sent for untargeted metabolomics. The abundance of 5-aminovalerate (B), proline (C) and glycine (D) in human stools samples derived from hospitalized controls (HC; N = 23) and CDI positive patients (N = 29) is quantified. Two-tailed Mann-Whitney test was used for group comparison: NS, not significant; ** p < 0.01; *** p < 0.0001.

In monoassociated animals, the Stickland substrates, proline and glycine (and prolylglycine), were lower in colonized mice suggesting that the amino acids were being consumed (S7 Fig). Conversely, the Stickland metabolic product, 5-aminovalerate, was significantly enriched (Fig 7A). Stickland metabolism is a major metabolic pathway for *C*. *difficile* [44–46]. In Stickland metabolism, amino acids are decarboxylated or deaminated, reducing NAD^+^ to NADH in the process [46]. In the reductive branch of the pathway, NAD^+^ is regenerated by the selenoproteins, proline reductase (PrdB) and glycine reductase (GrdA) [44–46]. In this process proline and glycine are consumed and 5-aminovalerate and acetate are generated, respectively. *C*. *scindens* and *C*. *hiranonis*, are predicted to encode the selenoprotein, PrdB, that produces 5-aminovalerate in the reductive branch of the Stickland pathway. Thus, it would be likely that 5-aminovalerate production would be high in both animal models that are resistant to CDI (*i*.*e*., *C*. *scindens*-produced) and in patients experiencing CDI (*i*.*e*., *C*. *difficile*-produced). Indeed, fecal 5-aminovalerate and glycine levels were significantly enriched in patients with CDI compared with hospitalized controls without diarrhea (Fig 7B), whereas the abundance of proline was not different between the two clinical groups (Fig 7C and 7D).

**Fig 8:**
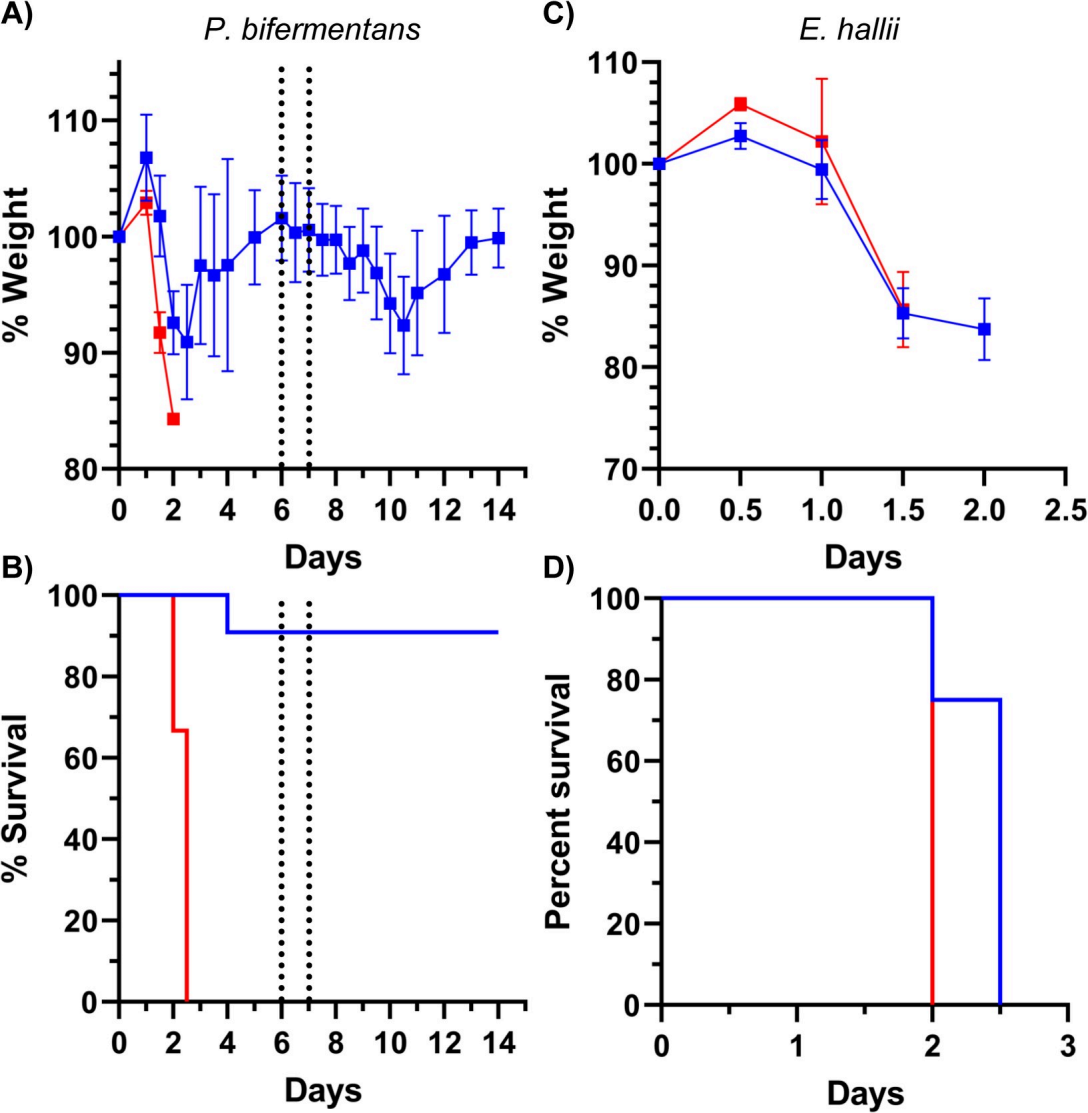
Stickland metabolism is important for colonization resistance. Spores derived from the *C*. *difficile* VPI10463 strain were inoculated into germ-free (N = 3) or mice monoassociated with (A) and (B) *P*. *bifermentans* (N = 11) or (C) and (D) *A*. *hallii* (N = 4). On day 6 post CDI, monoassociated mice were given a single i.p. dose of clindamycin to induce recurrence [39]. A and C represent the average weights of the infected mice. B and D represent the Kaplan-Meier survival.

To test our hypothesis that consumption of proline / glycine or production of 5-aminovalerate (*e*.*g*., through Stickland metabolism) leads to an environment that protects against CDI, we monoassociated germ-free, wildtype, mice with *Paraclostridium bifermentans* and infected these mice with *C*. *difficile* VPI10463 spores. Importantly, *P*. *bifermentans* cannot 7α-dehydroxylate bile acids but can perform Stickland metabolism [47]. *P*. *bifermentans*-colonized mice displayed mild disease when infected with *C*. *difficile* spores (Fig 8A). Upon a single dose of clindamycin to induce recurrence, these mice were completely protected against recurring disease (Fig 8B).

Finally, *Cyp8b1*^*-/-*^ mice were monoassociated with *Anaerobutyricum hallii*. *A*. *hallii* cannot 7α-dehydroxylate primary bile acids and does not encode enzymes required for Stickland metabolism (*i*.*e*., proline reductase or glycine reductase) and, thus, should not protect against CDI. *Cyp8b1*^*-/-*^ mice were monoassociated with *A*. *hallii* due to a prior report showing that this organism can aid the microbiome in producing secondary bile acids (specifically, taurodeoxycholic acid) and the *Cyp8b1*^*-/-*^ strain alleviates any potential effect of cholic acid-derivatives [48]. Indeed, *A*. *hallii*-colonized mice rapidly succumbed to *C*. *difficile* disease at a rate indistinguishable from germ-free controls (Fig 8C and 8D).

Taken together, our data indicate that, despite secondary bile acid production by a healthy microbiota being a useful biomarker for colonization resistance against *C*. *difficile* [12,26,31], secondary bile acid production and downstream FXR-FGF19 signaling does not appear to be the primary mechanism by which the microbiota protect the host against *C*. *difficile* colonization and infection.

## Discussion

CDI remains a major healthcare problem that is associated with poor antimicrobial stewardship leading to disruption of the gut microbiota and loss of 7α-dehydroxylating bacteria [12,14,26,29,31,49]. Germination of previously dormant *C*. *difficile* spores is considered the first step in pathogenesis. This step is potentiated, *in vitro*, by cholic acid class bile acids and amino acids. In a recent study, Leslie and colleagues demonstrated that consumption of the glycine cogerminant can block the colonization of *C*. *difficile* by reducing spore germination [20]. Though we find that *C*. *scindens*, *C*. *hiranonis* and *C*. *leptum* reduce the abundance of glycine, proline and prolylglycine in monoassociated mice (S7 Fig), we do observe *C*. *difficile* colonization but lack of disease (S3 Table). These results are similar to prior observations [26]. In prior work, colonization of mice with *C*. *scindens*, or a consortium of 4 bacteria, protected against CDI but still led to colonization of mice with *C*. *difficile* [26]. This suggests that in monoassociated mice, germination of dormant *C*. *difficile* spores still occurs but competition between the two strains prevents disease.

Prior work tested the importance of cholic acid recognition by *C*. *difficile* spores using a mutant *C*. *difficile* strain whose spores could not respond to cholic acid-class bile acids [8]. Infection of antibiotic treated hamsters with spores derived from the *C*. *difficile cspC*::*ermB* strain led to a reduction in disease to 50% of the infected hamsters [8]. With the generation of a cholic acid-deficient mouse strain, we tested if cholic acid production by the host is necessary for infection. Similar to the prior work, *Cyp8b1*^*-/-*^ mice still supported infection by *C*. *difficile* spores. Infection of these mice with spores derived from the *C*. *difficile cspC*::*ermB* mutant strain resulted in disease. Together, these results suggest that cholic acid-mediated spore germination enhances the efficiency of *in vivo* spore germination but a small amount of spores germinate in a bile acid independent manner and lead to colonization. Importantly, though, inhibition of bile acid-mediated germination can reduce CDI in animal models indicating that inhibitors of germination may overcome the *in vivo* autogermination of *C*. *difficile* spores [50–52].

Use of *C*. *difficile* targeting antibiotics to treat CDI leads to continued perturbation of the colonic microbiota and can contribute to recurrent disease. Despite the availability of anti-*C*. *difficile* antibiotics, the most effective treatment for recurrent and refractory disease is FMT. However, this experimental therapy is still the subject of intense FDA scrutiny because of the risk of other multidrug resistant infections [53]. To establish safer microbial treatment there therefore is a need to better understand how FMT provides clinical efficacy, currently assumed to be mediated by secondary bile acids reestablishing colonization resistance in the susceptible host [42]. This assumption is based on donor FMT preparations containing 7α-dehydroxylating bacteria which restore the balance of colonic secondary bile acid levels in patients and animal models. These largely correlative findings led to the hypothesis that secondary bile acid production by the colonic microbiota contributes to an environment that is toxic for *C*. *difficile* growth *in vivo* [34,35].

Our data suggest that 7α-dehydroxylation by the protective microbiota is not the primary mechanism by which these bacteria prevent infection. Deoxycholate is the major secondary bile acid in mice and is toxic to *C*. *difficile* growth *in vitro* [9,10]. A recent report tested the ability of different commensal strains to inhibit *C*. *difficile* growth [54]. The authors found that *C*. *scindens* efficiently inhibited *C*. *difficile* growth and this was correlated with high amounts of DCA. In this study, *C*. *hiranonis* did not inhibit *C*. *difficile* growth *in vitro* [54]. In the current study, monoassociation of germ-free mice with *C*. *scindens*, *C*. *hiranonis*, or *C*. *leptum* protected against CDI in the absence of secondary bile acid formation. Further, we demonstrate clinical translation of these reductionist models using human FMT donor microbiota preparations with proven efficacy in CDI patients, showing excellent protection in mice lacking cholic acid-class bile acids. Our collective data strongly supports a bile-acid independent mechanism of protection by 7α-dehydroxylating bacteria that does not involve previously reported CDI-directed antimicrobials.

Metabolomic profiling of patients and monoassociated germ-free mice led to our identification of Stickland metabolism as a likely contributor to protection against CDI, as previously reported [55]. Both *C*. *scindens* and *C*. *hiranonis* encode proline / glycine reductases but we could not find these orthologues in *C*. *leptum*. However, all 3 strains consume proline / glycine and produce 5-aminovalerate (Fig 7). Consumption of proline in Stickland metabolism generates 5-aminovalerate [45,46] production by the microbiota that may lead to either feedback inhibition of *C*. *difficile* Stickland metabolism or the consumption of proline by these bacteria would remove an essential amino acid for *C*. *difficile* Stickland metabolism. In the metabolomics dataset, we did not observe a decrease in most other amino acids (S4 Table). However, we did observe a lower abundance of threonine and serine. Interestingly, both of these are 1 enzymatic step from glycine and *C*. *difficile* encodes the enzymes that perform this reaction (threonine aldolase and serine hydroxymethyl transferase, respectively). We interpret this to mean that *C*. *difficile* consumes glycine, becomes starved for it and then converts Thr and Ser to glycine to continue Stickland. Alanine was moderately low and can be used in Stickland reactions to acetyl or propionyl-CoA [56]. Asparagine and glutamine were also lower (however not to the levels observed for Thr, Ser, Gly or Pro). These amino acids are good sources of nitrogen and we interpret this as such. Finally, valine was slightly reduced too. Valine can be consumed in the oxidative Stickland branch but the Stickland product, isobutyrate, was not present in the metabolomics dataset. This data contrasts with what was observed for amino acid presence in prior work [55]. However, this work did not use monoassociated studies and thus direct comparisons should not be made.

Building upon this, the same prior study found that in germ-free mice colonized with a consortia of bacteria derived from a dysbiotic gut, a *C*. *difficile* proline reductase mutant had difficulties in colonization, suggesting the importance of Stickland metabolism for *C*. *difficile* colonization [55]. In support of the hypothesis that Stickland metabolism (or the consumption of proline / glycine and / or production of 5-aminovalerate) is important for protecting against CDI, *P*. *bifermentans*-colonized mice (Stickland-positive, 7α-dehydroxylation-negative) were protected against CDI but *A*. *hallii*-colonized (Stickland-negative, 7α-dehydroxylation-negative) mice were not. Importantly, the production of 5-aminovalerate by *C*. *difficile* could result in these organisms being excluded from the diseased colon. Because our findings suggest that secondary bile acid generation by a healthy microbiome is not the mechanism by which colonization resistance is established, we propose that restoring Stickland metabolism (or bacteria that consume proline / glycine and / or produce 5-aminovalerate independently of Stickland) to the colonic microbiome should be a focus of ongoing efforts to re-establish colonization resistance in susceptible hosts and that this should be investigated further in a larger cohort of patients.

## Materials and methods

### Ethics statement

Ethical approval for the study was obtained from the Liverpool Research Ethics Committee under reference numbers 08/H1005/32 and each patient provided written informed consent prior to recruitment. Ethical approval for omics screening of patients was also granted by Baylor College of Medicine and University of Houston Institutional Review Boards.

All animal studies were performed with prior approval from the Texas A&M University Institutional Animal Care and Use Committee (IACUC# 2020–0025) and from the Baylor University College of Medicine Institutional Animal Care and Use Committee (IACUC# AN-914).

### Growth conditions

*C*. *scindens* VPI12708, *C*. *hiranonis* 10542, *C*. *leptum* ATCC 29065, *P*. *bifermentans* ERIN_30100, and *A*. *hallii* DSM3353 were grown on BHI medium (Brain heart infusion) until reaching logarithmic phase. *C*. *difficile* was grown in BHIS medium for six hours. (Brain heart infusion supplemented with 5 g / L yeast extract). All clostridial strains were grown in an anaerobic environment (Model B, Coy Laboratories Grass Lake, MI) at 37°C (85% N_2_, >3% H_2_, and 5% CO_2_).

### Deconjugation/dehydroxylation assay

*C*. *scindens* VPI12708 and *C*. *hiranonis* 10542 were grown on BHI medium for 16 hours. Cultures were back diluted to 10^7^ and a 1:10 dilution was added to BHI medium supplemented with 1 mM taurocholate. Cultures were grown for 24 hours and then centrifuged for 5 minutes at 4000 x g. Samples were then lyophilized. The presence of specific bile acids in samples was measured as described below.

### Bile acid extraction and separation

Bile acids were extracted from 200 mg lyophilized stool, as described previously [57]. The extracted bile acids were separated by reverse-phase HPLC using a Shimadzu prominence HPLC system. Twenty-five microliter samples were separated using a Synchronis C18 column (4.6 by 250 mm; 5 μm particle size; ThermoFisher 97105–254630) using a mobile phase consisting of 53% methanol, 24% acetonitrile, 23% water and 30 mM ammonium acetate (pH 5.6) [58]. Bile acid peaks were detected using a Sedere Sedex model 80 LT- ELSD (low temperature-evaporative light scattering detector) using an air pressure of 50 psi of Zero Grade air at 94°C [58,59]. Different amounts of specific bile acids [taurocholic acid (Sigma Aldrich 86339-25G), glycocholic acid (Sigma Aldrich G7132-1G), taurochenodeoxycholic acid (Sigma Aldrich T6260-250MG), glycochenodeoxycholic acid (Sigma Aldrich G0759-500MG), hyodeoxycholic acid (Sigma Aldrich H3878-5G), chenodeoxycholic acid (Acros organics C9377-25G), cholic acid (Sigma Aldrich C1129-100G), deoxycholic acid (Sigma Aldrich D2510-100G), lithocholic acid (Acros organics L6250-5G), α-muricholic acid (Steraloids C1890-000), β-muricholic acid (Steraloids C1895-000), ω-muricholic acid (Steraloids C1888-000)] were separated as described above to generate standard curves. The area under each peak was calculated and plotted against the concentration of bile acid added and a trend line was generated for each bile acid. A 10 nmol injection of all bile acids used on this study was done to generate the bile acid chromatograph shown in S3 Fig. To specifically identify bile acids in the extracted samples, aliquots of bile acid extractions were spiked with standards to assign peaks of specific bile acids. Concentration of the bile acids in samples (nmol) was calculated using the standard curves of pure bile acids and normalized with the added internal standard (HDCA) [59]. Bile acid concentrations were divided by weight of sample used for extraction to calculate concentration per gram of sample.

### Quantitative PCR

Amplification and detection of specific DNA targets for *C*. *difficile* and *C*. *scindens* was performed on QuantStudio Flex real-time PCR system. All samples were run in triplicate in fast 96-well plates (Applied biosystems 4346907). The PCR reaction was performed on a 10 μL volume. Species-specific genes were tested and selected for each bacterial species. The *baiE* gene was used for *C*. *scindens* and the *tcdA* gene was used for *C*. *difficile*. Samples were normalized to a concentration of 100 ng / μL; 1 μL per sample was used in the reaction along with 5 mM forward and reverse oligonucleotides. PowerUP SYBR Green master mix (Thermo Scientific, A25742) was used.

### Minimum inhibitory concentration of deoxycholate and lithocholate

The minimal inhibitory concentrations of DCA and LCA were determined using standard techniques. Cultures of *C*. *difficile* VPI10463 were grown overnight. The following day, cultures were back diluted and grown until reaching an OD_600_ = 0.5 before inoculating into a range of DCA / LCA concentrations. The minimum inhibitory concentration was determined at the lowest dilution of DCA or LCA where *C*. *difficile* did not grow.

### Mice experiments

The CYP8B1 mouse mouse C57BL/6N-*Cyp8b1*^*tm1(KOMP)Vlcg*^/MbpMmucd was purchased from the Mutant Mouse Resource & Research Center at UC-Davis (Stock No. 047289-UCD). Heterozygous mice were rederived to germ-free status by the Baylor College of Medicine Gnotobiotics Core using the hysterectomy/cross-foster method. Mice were confirmed germ-free by serial negative 16S qPCR and aerobic, anaerobic, and fungal culture over a 90 day period. Germ-free mouse colonies were housed in sterile flexible film isolators (Class Biologically Clean, Madison, WI). Monoassociated mice were gavaged with liquid cultures of *C*. *scindens*, *C*. *hiranonis*, *C*. *leptum*, *P*. *bifermentans* or *A*. *hallii* at 10^6^ CFU / mL and housed in positive pressure individually ventilated cages (IsoCage P, Tecniplast, Buguggiate, Italy). Colonization was confirmed with 16S PCR. Stably colonized mice or GF control mice were infected with 10^4^ *C*. *difficile* VPI10463 spores, or 10^6^ *C*. *difficile* UK1 or JSC10 (*cspC*::*ermB*) spores (inoculum was increased compared to VPI10463 due to reduced virulence of the UK1 strain compared to the VPI10463 strain) [39]. Infected mice were monitored for weight loss and any clinical signs of disease. When stated, an intraperitoneal injection of clindamycin (10 mg / kg) was administered to test for disease recurrence. All animals were handled in accordance with the protocols approved by the Institutional Animal Care and Use Committees at Texas A&M University and at Baylor College of Medicine.

### Microbiome sequencing and analysis

Amplicon data of 16S rDNA variable region of V4 was generated from MiSeq platform with paired-end sequencing protocol (effective read length: 250 bp for V4). DADA2 package (version 1.8) [60] was used for processing de-multiplexed raw sequencing reads following the default parameter settings with minor modifications. Specifically, raw sequencing reads were trimmed while maintaining the overlap regions for merging paired-end reads by VSEARCH (version 2.9) [61]; sequencing primers in paired-end reads were stripped by the length of primers. IdTaxa function in DECIPHER package (version 2.6.0) [62] and its pre-built training set (LearnTaxa function) of SILVA database (release 132) [63] was used for taxonomy assignment (threshold: 0.5) of amplicon sequence variants (ASVs) from DADA2 output. ASVs that were not classified as bacteria domain and that were identified as chimeras were removed. Samples with less than 4,500 reads in the ASV matrix (median read count: 34,093) were excluded and proportional transformation was applied to normalize feature data prior to downstream analysis. Alpha-diversity indices including Shannon metric were calculated by Phyloseq package (version 1.24.2) [64]. Beta-diversity analysis was performed with non-metric multidimensional scaling (NMDS) of Bray-Curtis dissimilarity distance metric in the Vegan package (version 2.5–5) [65]. Non-parametric statistical test using ANalysis Of SIMilarities (ANOSIM) method in vegan was used for comparing group difference using beta-diversity distance matrix. Raw reads for the microbiome studies have been deposited in the sequence read archive at NCBI (Accession number: PRJNA726993)

### Patient cohort

Patients and age matched hospitalized controls (HC) were recruited from a large hospital setting in Merseyside, UK. Consecutive patients with healthcare-associated diarrhea, which was defined as ≥3 liquid stools passed per day in the 24 hours preceding assessment, an onset after being in hospital for over 48 hours and recent exposure to either antimicrobials and/or proton pump inhibitors, were eligible for inclusion. Relevant information on demographics, admission and clinical evaluation was collected for each patient who consented to participate and recorded into an anonymised case report proforma. Patients were identified by a daily review of *C*. *difficile* ELISA toxin tests performed by the clinical microbiology laboratories. Clinical metadata collected on all patients included demographics (age and sex), comorbid conditions (Charlson comorbidity index), use of antibiotics, and other medications. Fecal metabolome analysis was performed on 29 CDI patients and 23 age matched hospitalized control (HC) subjects. Serum FGF19 levels was measured in fasted, early morning blood draws in age matched CDI patients (n = 29) and HC (n = 75). In order to minimize bias in reporting assay results, specimens were assigned non-identifiable codes that were decoded after study completion.

### FGF19 measurement and stool metabolome analysis

Fibroblast Growth Factor 19 (FGF19) from human serum samples was measured by ELISA (R&D Systems). Human stool samples were sent to Metabolon inc. for untargeted metabolomics analysis. Raw intensity values of metabolites from Metabolon were transformed with log10 scaling for downstream analysis, and missing values (non-detected) were imputed with zero. Only primary and secondary bile acids and amino acids were included to meet the scope of this study. Heatmap was used to visualize the transformed data between CDI and HC subjects. Median absolute deviation, median, mean and standard deviation of each metabolite in each group were calculated with base functions in R package. Benjamini-Hochberg (BH) correction was applied for statistical analysis with two-sided Mann-Whitney-Wilcoxon test for group comparison.

## Supporting information

S1 Fig Model for bile acid-mediated protection against CDI. A) In a healthy colonic environment, the microbiome is hypothesized to inhibit *C*. *difficile* growth through the production of secondary bile acids [*e*.*g*., deoxycholate (DCA)], an activator of *C*. *difficile* spore germination but a potent inhibitor of *in vitro C*. *difficile* growth). B) In a dysbiotic colonic environment, *C*. *difficile* spore germination is thought to be triggered by the combinatorial action of cholic (CA) acid-class bile salts [*e*.*g*., taurocholate (TA)] and amino acids. Dormant spores white circles, germinated spores; dark circles, vegetative cells; rectangles.
(TIF)

S2 Fig Confirmation of the *baiE* orthologue. The presence of a *baiE* orthologue was confirmed by PCR using oligonucleotides specific for each strain.
(TIF)

S3 Fig Detection of bile acid standards using HPLC. The indicated bile acids were separated by HPLC and detected using evaporative light scattering. A standard curve was generated using this method and used to quantitate the bile acids.
(TIF)

S4 Fig Bile salt hydrolase activity in *C*. *scindens* and *C*. *hiranonis*. The indicated strains were grown for 24 hours in medium supplemented with taurocholate. The abundance of taurocholate or cholate in the media fraction of the culture was determined by HPLC and expressed as a percentage of the total input. Bars represent the average from three independent experiments and error bars are the standard error of the mean. *C*. *scindens* generated no cholate in the experiments.
(TIF)

S5 Fig FXR^-/-^ are as susceptible as wildtype mice to *C*. *difficile* infection. Antibiotic-treated wildtype (N = 7) and FXR^-/-^ (N = 11) were infected with *C*. *difficile* VPI10463 spores. Animals were monitored for disease symptoms and weighed daily for two days. Differences in weight loss are non-significant.
(TIF)

S6 Fig Bile acid, FGF19 and age of CDI patients and hospitalized controls. Stool primary and secondary bile acids were profiled by Metabolon for 29 CDI patients and 23 hospitalized control (HC) subjects. Serum FGF19 level was measured by ELISA for the same 29 CDI patients and another 75 HC subjects of the same cohort. Two-tailed Mann-Whitney test was used for group comparison: NS, not significant.
(TIF)

S7 Fig Abundance of proline, glycine and prolylglycine in the untargeted metabolomics. Cecal contents from germ-free, conventionally-raised, or monoassociated mice were sent for untargeted metabolomics and lipodomics. The abundance of A) proline, B) glycine and C) the dipeptide, prolylglycine, is illustrated.
(TIF)

S1 Table Minimal Inhibitory Concentration of secondary bile acids on *C*. *difficile* VPI10463. (DOCX)

S2 Table Bile acid amounts in germ-free, *C*. *scindens*-, or *C*. *hiranonis*-, or *C*. *leptum-*colonized mice. (DOCX)

S3 Table *C*. *scindens* and *C*. *difficile* colonization levels of *Cyp8b1* pre- and post-infection. (DOCX)

S4 Table Full metabolomics dataset. (XLSX)
