# Supplementary figures and images for: Bile acid-independent protection against Clostridioides difficile infection
Source: PLoS Pathog. 2021 Oct 19;17(10):e1010015. doi: 10.1371/journal.ppat.1010015 (PMC8555850; doi:10.1371/journal.ppat.1010015)

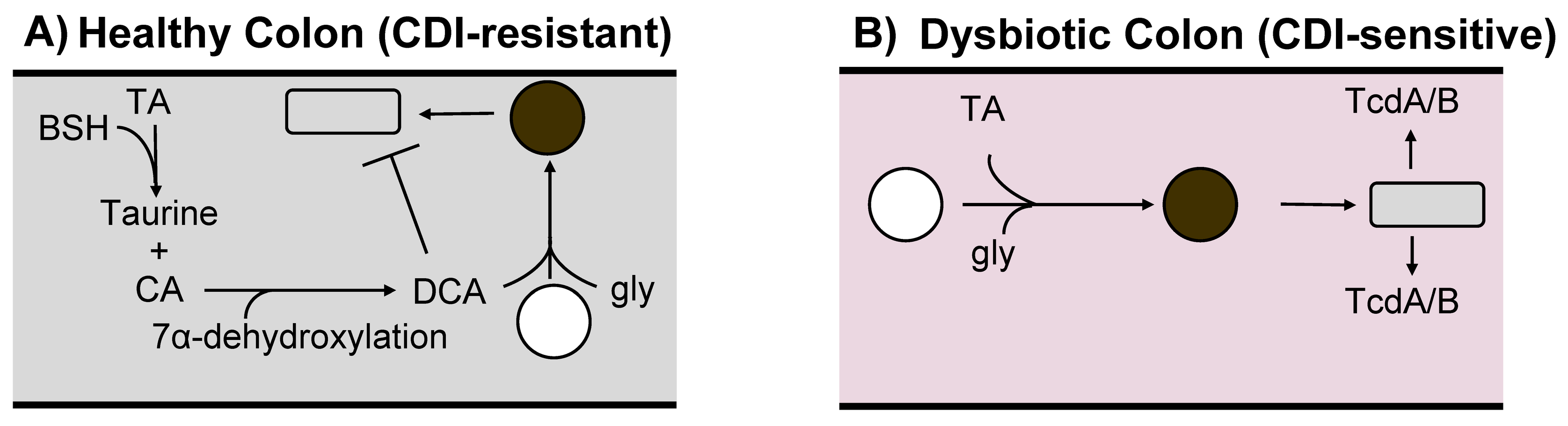

Supplement: S1 Fig — A) In a healthy colonic environment, the microbiome is hypothesized to inhibit C. difficile growth through the production of secondary bile acids [e.g., deoxycholate (DCA)], an activator of C. difficile spore germination but a potent inhibitor of in vitro C. difficile growth). B) In a dysbiotic colonic environment, C. difficile spore germination is thought to be triggered by the combinatorial action of cholic (CA) acid-class bile salts [e.g., taurocholate (TA)] and amino acids. Dormant spores white circles, germinated spores; dark circles, vegetative cells; rectangles. (TIF) [file ppat.1010015.s001.tif]

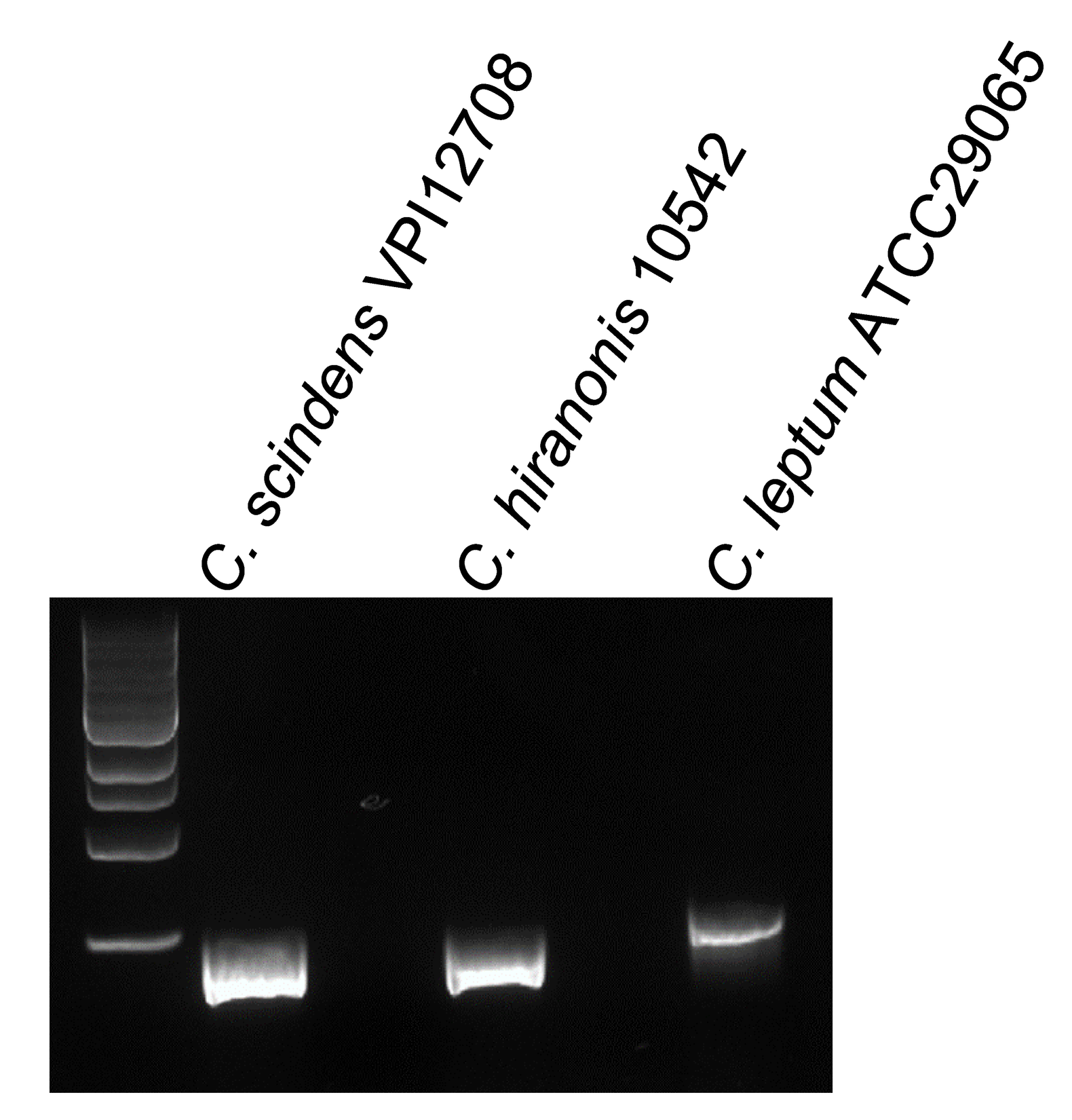

Supplement: S2 Fig — The presence of a baiE orthologue was confirmed by PCR using oligonucleotides specific for each strain. (TIF) [file ppat.1010015.s002.tif]

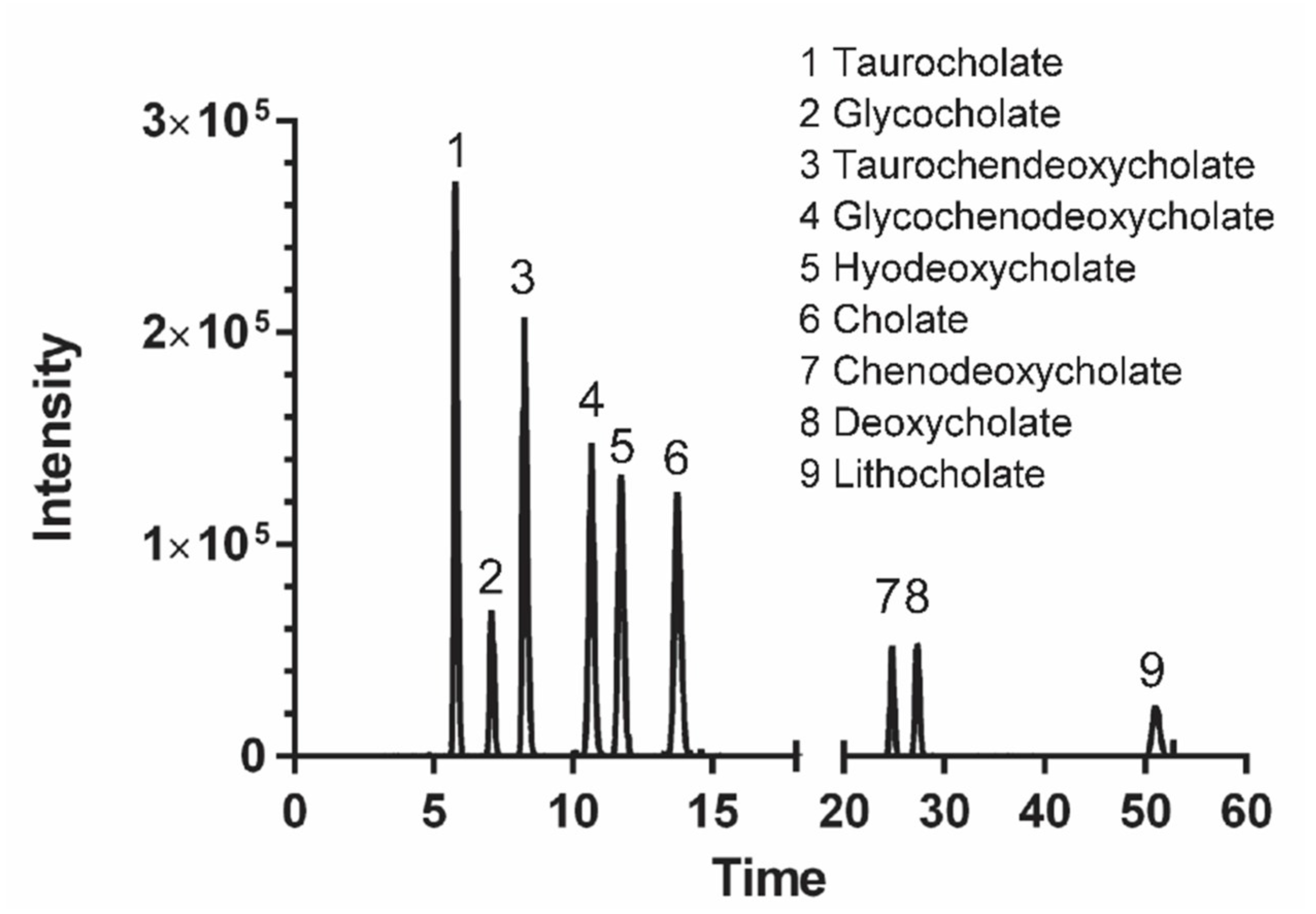

Supplement: S3 Fig — The indicated bile acids were separated by HPLC and detected using evaporative light scattering. A standard curve was generated using this method and used to quantitate the bile acids. (TIF) [file ppat.1010015.s003.tif]

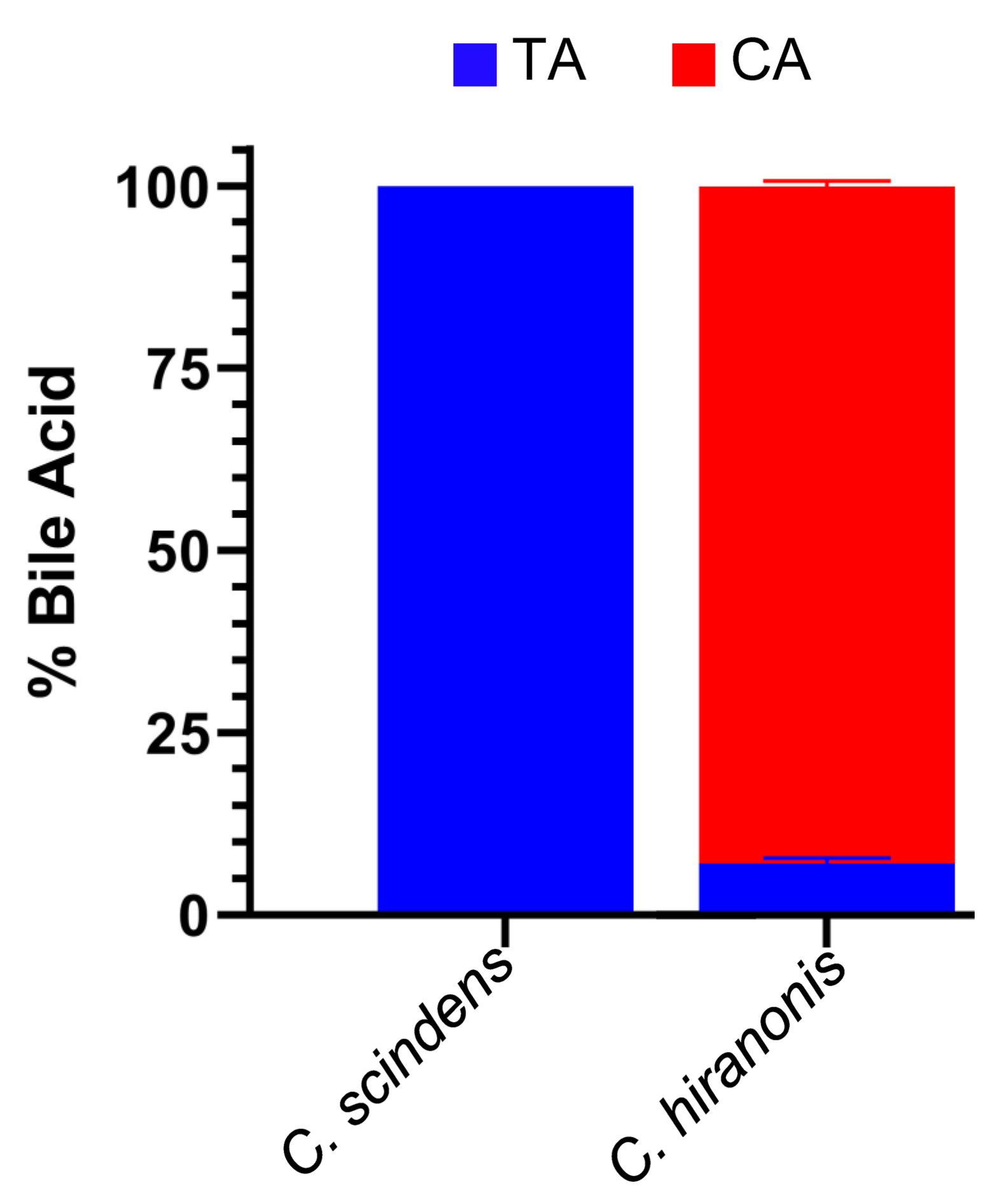

Supplement: S4 Fig — The indicated strains were grown for 24 hours in medium supplemented with taurocholate. The abundance of taurocholate or cholate in the media fraction of the culture was determined by HPLC and expressed as a percentage of the total input. Bars represent the average from three independent experiments and error bars are the standard error of the mean. C. scindens generated no cholate in the experiments. (TIF) [file ppat.1010015.s004.tif]

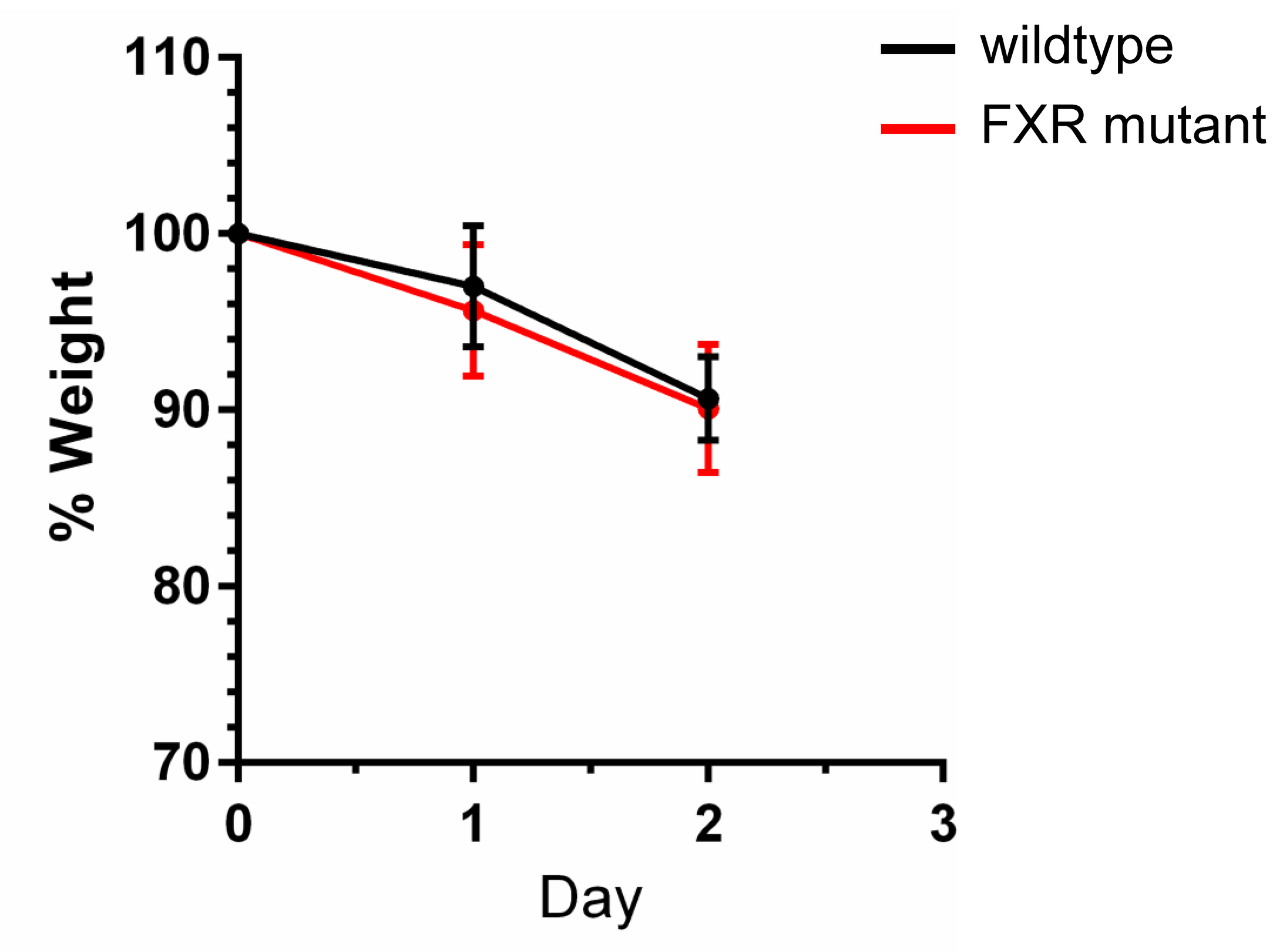

Supplement: S5 Fig — Antibiotic-treated wildtype (N = 7) and FXR-/- (N = 11) were infected with C. difficile VPI10463 spores. Animals were monitored for disease symptoms and weighed daily for two days. Differences in weight loss are non-significant. (TIF) [file ppat.1010015.s005.tif]

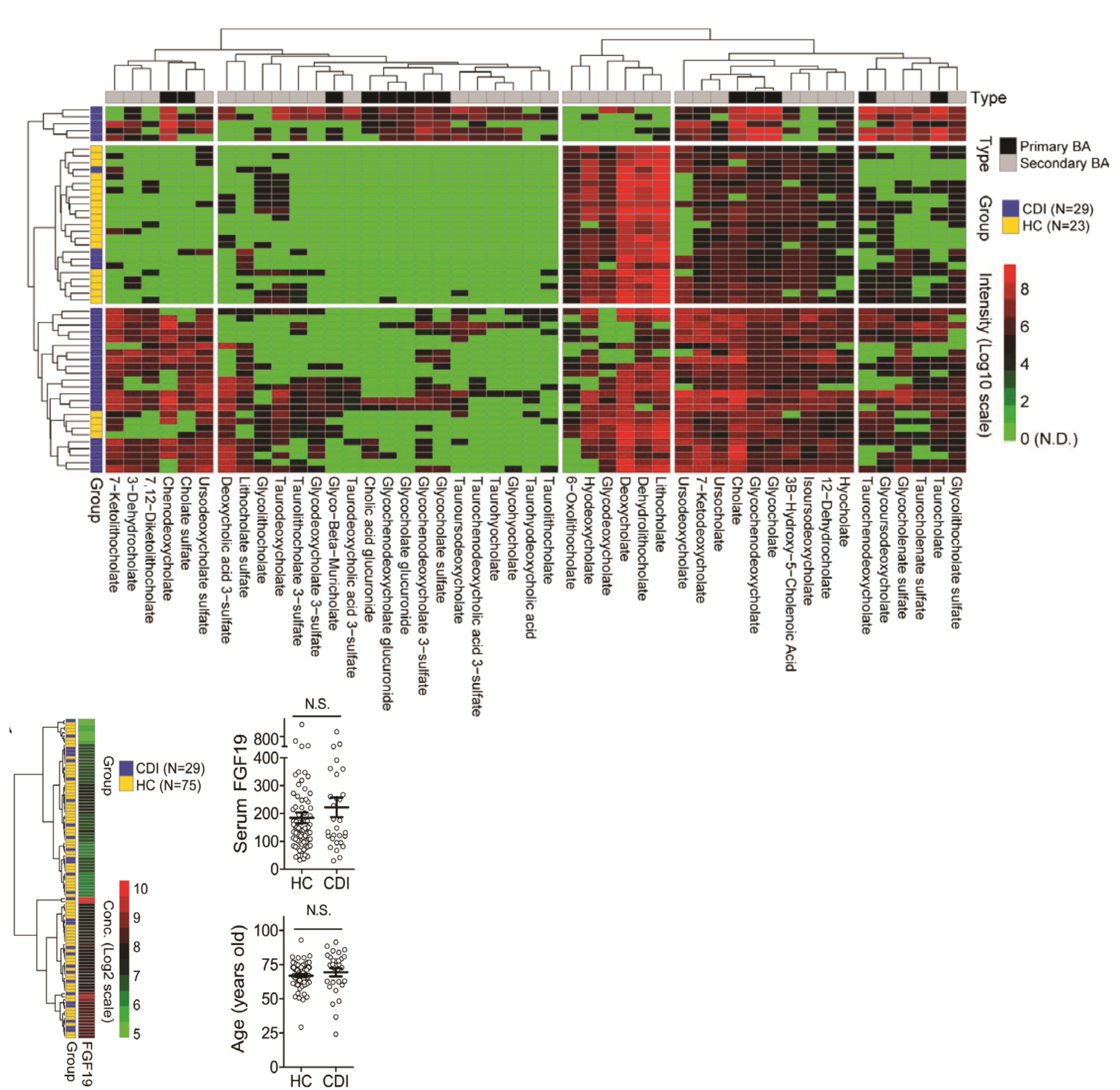

Supplement: S6 Fig — Stool primary and secondary bile acids were profiled by Metabolon for 29 CDI patients and 23 hospitalized control (HC) subjects. Serum FGF19 level was measured by ELISA for the same 29 CDI patients and another 75 HC subjects of the same cohort. Two-tailed Mann-Whitney test was used for group comparison: NS, not significant. (TIF) [file ppat.1010015.s006.tif]

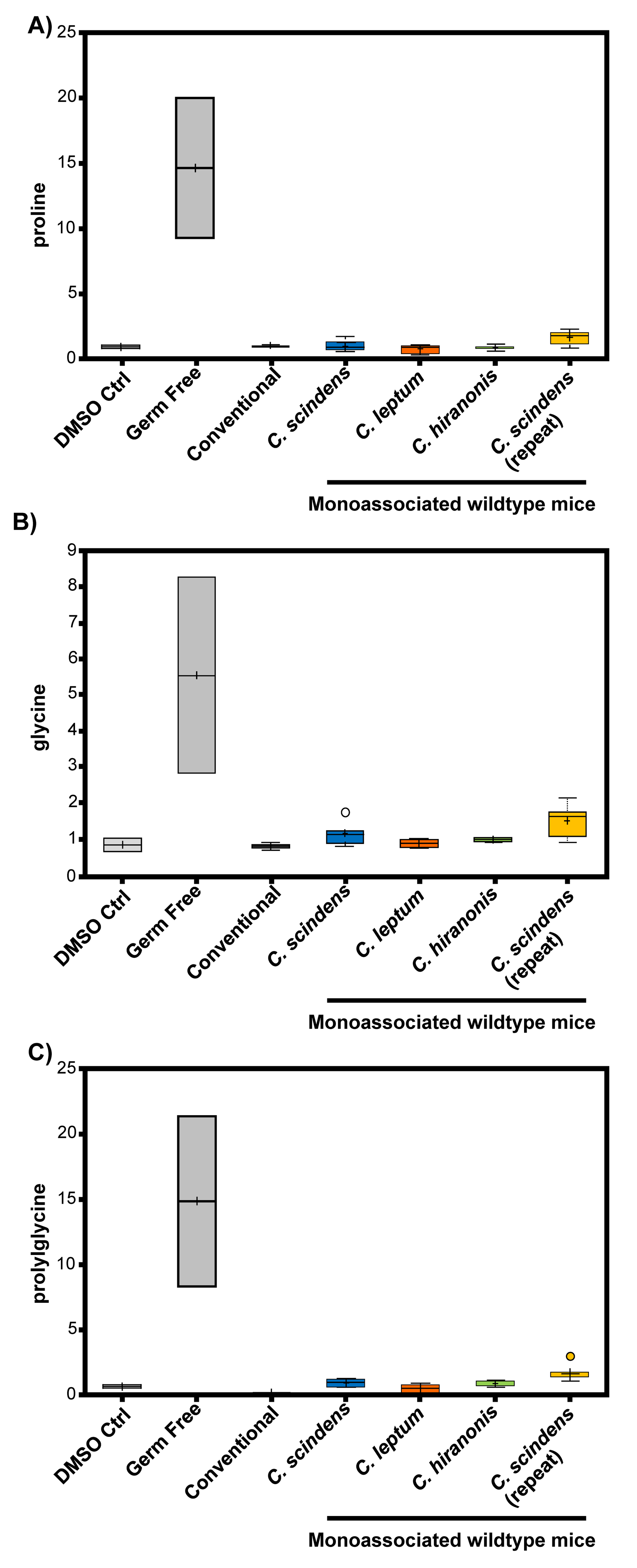

Supplement: S7 Fig — Cecal contents from germ-free, conventionally-raised, or monoassociated mice were sent for untargeted metabolomics and lipodomics. The abundance of A) proline, B) glycine and C) the dipeptide, prolylglycine, is illustrated. (TIF) [file ppat.1010015.s007.tif]
