# Supplementary material for: Bile acid-independent protection against Clostridioides difficile infection
Source: PLoS Pathog. 2021 Oct 19;17(10):e1010015. doi: 10.1371/journal.ppat.1010015 (PMC8555850; doi:10.1371/journal.ppat.1010015)
Supplement: S1 Table — (DOCX) [file ppat.1010015.s008.docx]

**S1 Table. Minimal Inhibitory Concentration of secondary bile acids on *C. difficile* VPI10463**

|  | *C. difficile* VPI10463 | |
| --- | --- | --- |
|  | Deoxycholate | Lithocholate |
| No solvent | 1 mM | NA |
| 4% ethanol | 0.6 mM | 0.5 mM |

NA: Lithocholate was not soluble unless 4% ethanol was included in the culture medium. Both the presence of and absence of 4% ethanol are shown for comparison. Experiments were performed in triplicate, the data did not vary between experiments.
