# Supplementary material for: Bile acid-independent protection against Clostridioides difficile infection
Source: PLoS Pathog. 2021 Oct 19;17(10):e1010015. doi: 10.1371/journal.ppat.1010015 (PMC8555850; doi:10.1371/journal.ppat.1010015)
Supplement: S2 Table — (DOCX) [file ppat.1010015.s009.docx]

**S2 Table. Bile acid amounts in germ free, *C. scindens*-, or *C. hiranonis*-, or *C. leptum-*colonized mice**.

|  | Concentration (nmol / g) | | | | |
| --- | --- | --- | --- | --- | --- |
| Colonization status | TA | CA | AMA | DCA | OMA |
| Germ free | 19.9 | - | - | - | - |
| Germ free | 5.9 | - | - | - | - |
| Germ free | 37.0 | - | - | - | - |
| Germ free | 30.9 | - | - | - | - |
| *C. hiranonis* | 2.7 | - | - | - | 4.0 |
| *C. hiranonis* | 23.6 | 25.3 | - | - | 3.8 |
| *C. leptum* | 19.2 |  | 0.4 | - | - |
| *C. leptum* | 7.3 | - | - | - | - |
| *C. scindens* | 46.0 | - | - | - | 3.6 |
| *C. scindens* | 67.2 | - | 0.3 | 18.5 | 6.3 |
| *C. scindens* | 56.6 | - | 1.6 |  | 5.8 |

- **taurocholate (TA), cholate (CA), alpha-muricholate (AMA), deoxycholate (DCA), omega-muricholate (OMA).**
- Below limit of detection
- Limit of detection for Sedere Sedex model 80 LT- ELSD was calculated to be 0.2 nmol.

GCA, TCDCA, GCDCA, CDCA & BMA were all below the limit of detection
