# Supplementary material for: Bile acid-independent protection against Clostridioides difficile infection
Source: PLoS Pathog. 2021 Oct 19;17(10):e1010015. doi: 10.1371/journal.ppat.1010015 (PMC8555850; doi:10.1371/journal.ppat.1010015)
Supplement: S3 Table — (DOCX) [file ppat.1010015.s010.docx]

**S3 Table. *C. scindens* and *C. difficile* colonization levels of *Cyp8b1* pre- and post-infection.**

|  | Gene copy number / g | |
| --- | --- | --- |
| **Genotype** | **baiE** | **tcdA** |
| Pre-infection | | |
| HOM | 9.19E+05 ± 2.7E+04 |  |
| HOM | 8.02E+06 ± 6.7E+04 |  |
| HET | 1.24E+07 ± 9.3E+05 |  |
| HET | 1.98E+07 ±6.9E+05 |  |
| Post-infection | | |
| HOM | 8.45E+03 ± 6.3E+02 | 5.00E+05 ± 1.4E+04 |
| HOM | 5.63E+03 ± 5.6E+02 | 1.03E+06 ± 5.3E+04 |
| HOM | 7.54E+04 ± 3.6E+03 | 7.95E+05 ± 8.5E+04 |
| HOM | 2.96E+03 ± 4.4E+02 | 3.64E+05 ± 4.4E+04 |
| HET | 1.25E+05 ±1.9E+04 | 1.67E+06 ± 8.6E+04 |
| HET | 2.12E+05 ± 3.2E+04 | 8.76E+06 ± 7.7E+05 |
| HET | 9.7E+04 ±3.6E+03 | 1.24E+06 ± 3.1E+ 04 |
| WT | 2.82E+03 ± 1.2E+02 | 1.19E+06 ± 1.4E+04 |
